# CMV chemotherapy for advanced transitional cell carcinoma.

**DOI:** 10.1038/bjc.1992.310

**Published:** 1992-09

**Authors:** G. M. Jeffery, G. M. Mead

**Affiliations:** CRC Wessex Regional Medical Oncology Unit, Southampton General Hospital, UK.

## Abstract

Between May 1986 and September 1990 a total of 43 patients with metastatic transitional cell carcinoma (TCC) of the urinary tract have been treated at our institution with combination chemotherapy (CMV) consisting of cisplatin 100 mg m-2 i.v. day 2; methotrexate 30 mg m-2 i.v. days 1.8; and vinblastine 4 mg m-2 i.v. days 1.8. Chemotherapy was recycled on day 22 and continued for a maximum of six cycles in responding patients. Of 33 patients with measurable disease 8 (24%) achieved a complete remission (CR). The median survival for patients achieving a CR was 13 months (range 5-29+) whilst the median survival for all 43 patients was 7 months (range 1-29+). Only three patients are still alive--two are disease free. More effective and/or less toxic chemotherapy regimens are needed for the treatment of patients with metastatic TCC.


Br. J. Cancer (1992), 66, 542-546 © Macmillan Press Ltd., 1992

### CMV chemotherapy for advanced transitional cell carcinoma

G.M. Jeffery & G.M. Mead

*CRC Wessex Regional Medical Oncology Unit, CF99, Southampton General Hospital Southampton SO9 4XY, UK.*

**Summary** Between May 1986 and September 1990 a total of 43 patients with metastatic transitional cell carcinoma (TCC) of the urinary tract have been treated at our institution with combination chemotherapy (CMV) consisting of cisplatin 100 mg $m^{-2}$ IV day 2; methotrexate 30 mg $m^{-2}$ IV days 1,8; and vinblastine 4 mg $m^{-2}$ IV days 1,8. Chemotherapy was recycled on day 22 and continued for a maximum of six cycles in responding patients. Of 33 patients with measurable disease 8 (24%) achieved a complete remission (CR). The median survival for patients achieving a CR was 13 months (range 5–29 +) whilst the median survival for all 43 patients was 7 months (range 1–29 +). Only three patients are still alive – two are disease free. More effective and/or less toxic chemotherapy regimens are needed for the treatment of patients with metastatic TCC.

Patients with metastatic transitional cell carcinoma (TCC) have a median survival of 3 months if untreated (Babaian *et al.*, 1980). During the 1970's a number of cytotoxic agents were investigated for their efficacy in treating patients with advanced TCC. When used as single agents overall response rates of between 16 and 35% were consistently reported for cisplatin, methotrexate, cyclophosphamide, doxorubicin, 5-fluorouracil, and vinblastine (Yagoda *et al.*, 1980). However complete responses were rare and response durations brief.

In 1981 investigators from the Northern California Oncology Group (NCOG) began a phase II study in patients with metastatic transitional cell carcinoma of a chemotherapy combination comprising cisplatin, methotrexate and vinblastine (CMV). They reported on the first 60 patients entered into this study in 1985 (Harker *et al.*, 1985) and documented a complete response rate of 28% with an overall median survival of 8 months. In 1983 the Memorial Hospital in New York began treating patients with advanced TCC with M-VAC combination chemotherapy which incorporated four of the most active single agents – methotrexate, vinblastine, doxorubicin and cisplatin. They reported their initial series in 1985 (Sternberg *et al.*, 1985) and have since added to and updated their experience (Sternberg *et al.*, 1988; Sternberg *et al.*, 1989). They have consistently reported encouraging clinical complete response rates of between 26% and 48% and in their most recent report the median survival for the whole group was 13 months with an estimated probability of 4-year survival of 18% +/− 7% (Sternberg *et al.*, 1989). Both the NCOG and the Memorial groups incorporated surgical resection of residual disease, where feasible, into their treatment regimens.

Between May 1986 and September 1990 43 patients with advanced TCC of the urinary tract have been treated with CMV chemotherapy at the CRC Wessex Regional Medical Oncology Unit in Southampton. These patients form the basis of this report.

### Material and methods

The characteristics of the 43 patients are summarised in Table I. All patients had biopsy proven transitional cell carcinoma of the bladder (33), renal pelvis (five), ureter (three or prostate (two). Three patients had tumours with mixed histology – two with TCC and adenocarcinoma (both prostatic primaries) and 1 with TCC and squamous cell carcinoma (bladder primary). There were 35 males and eight females and the median age was 65 years (range 46–75). During this period ten other patients with advanced TCC were treated with non-CMV chemotherapy regimens – in most cases because of impaired renal function. Patients treated with chemotherapy were highly selected as advanced age or serious co-existing medical conditions were regarded as contraindications to this form of therapy.

**Table I** Patient characteristics

| | | | |
|---|---|---|---|
| *Sex:* | Male | | – 35 (81%) |
| | Female | | – 8 (19%) |
| *Primary site:* | Bladder | | – 33 (77%) |
| | Renal pelvis | | – 5 (12%) |
| | Ureter | | – 3 (7%) |
| | Prostate | | – 2 (4%) |
| *Previous treatment:* | | | |
| | Radiotherapy (RT) | | – 23 (54%) |
| | Surgery | | – 12 (28%) |
| | None | | – 4 (9%) |
| | Surgery + RT | | – 4 (9%) |
| *Disease sites:* | | | |
| (i) | Parenchymal (20 patients): | | |
| | | Bone | – 13 |
| | | Liver | – 8 |
| | | Lung | – 7 |
| (ii) | Nodal (17 patients); | | |
| | | Abdominal | – 17 |
| | | Pelvic | – 15 |
| | | Cervical | – 4 |
| (iii) | Locally advanced (6 patients): | | |

Three patients presented with metastatic disease and were treated with primary chemotherapy. The remaining patients had received initial treatment for their primary disease. Twenty-three patients had received radiotherapy to the bladder (a median of 8 months earlier), and 12 patients had undergone either cystectomy or nephrectomy (a median of 4 months earlier). Three patients had surgery and radiotherapy for their primary tumour.

Six patients had presented following treatment of the primary with inoperable locally extensive disease in the pelvis. Seventeen patients had nodal metastases (pelvic, abdominal, cervical) only and 20 patients had metastatic involvement of bone, lung or liver. One patient received CMV chemotherapy as a result of histologically positive resection margins at surgery. Thirty-three (77%) of the patients had disease which was measurable using standard criteria (determined radiologically). No patient had received

Correspondence: G.M. Mead.
Received 12 December 1991; and in revised form 24 April 1992.

prior systemic chemotherapy.

All patients were assessed by physical examination, full blood count, biochemistry screen, liver function tests, chest x-ray and abdominal ultrasound. In addition most patients underwent computerised tomographic (CT) scanning of the abdomen and pelvis; isotope bone scanning was performed in patients with skeletal symptoms.

All patients had leukocyte counts greater than $3.5 \times 10^9 \, l^{-1}$ neutrophil counts greater than $2.0 \times 10^9 l^{-1}$ and platelet counts greater than $150 \times 10^{9 \; -1}$ at the time of commencing chemotherapy. The creatinine clearance was calculated according to the formula of Cockcroft and Gault (1976) and only patients with a creatinine clearance of greater than $50 \, ml \, min^{-1}$ were eligible to receive CMV chemotherapy. Patients with hydronephrosis and levels of renal impairment precluding CMV chemotherapy had nephrostomy tubes inserted in an effort to improve renal function and make them eligible for CMV treatment.

Patients were treated as outlined in Table II. The day 1 and 8 treatments were given as an outpatient and on day 2 patients were admitted to hospital and prehydrated with intravenous normal saline. When urine flow was greater than $150 \, ml \, h^{-1}$ cisplatin was given by 30 min infusion. Treatment was recycled on day 22 for a total of six cycles unless there was evidence of progressive disease or intolerable treatment side-effects. Drug doses were modified according to blood counts and renal function as outlined in Table II. Seven patients at the end of the study period received cisplatin at a dose of $70 \, mg \, m^{-2}$ from the outset in an attempt to limit cisplatin toxicity. Only one patient underwent post-chemotherapy surgery, at which a retroperitoneal mass was resected. No residual tumour was apparent – despite this the patient relapsed again at this site.

A complete response was defined as complete resolution of all radiological and clinical evidence of disease as assessed 1 month after the completion of chemotherapy. Partial response was defined as a 50% or greater reduction in the diameter of all tumour masses evident clinically and radiologically and was the best response recorded at any time during or immediately after chemotherapy.

Survival was calculated from the date of first chemotherapy until the date of last follow-up or death.

### Results

Thirty-three patients had measurable disease. Of the remaining ten patients, six had pelvic recurrence visualised on CT which which was not measurable, three had bone metastases and no other measurable disease and one patient received chemotherapy for histologically positive surgical resection margins after cystectomy. Eight of the patients with measurable disease achieved a complete response (24%) and 11 patients achieved a partial response (34%) for an overall response rate of 58%. One of the three patients with bone metastases as the only metastatic site had complete resolution of all symptoms and return to normal of the alkaline phosphatase level. Of the 33 patients with bladder primaries five (15%) achieved CR compared with three (30%) out of ten patients with non-bladder primaries. Six (35%) out of 17 patients with disease confined to nodal metastatic sites achieved CR compared with only two (12%) out of 17 patients with measurable parenchymal (lung, liver, bone) metastases. The median duration of CR was 6 months (range 1–20 + months).

The median survival duration for patients achieving CR was 13 months (range 5–29 + months). Three patients are still alive, however only two are progression free 19 and 27 months after completing chemotherapy.

Figure 1 shows the survival curve of all 43 patients receiving CMV chemotherapy. Figure 2 illustrates the survival of patients grouped according to whether their metastases were nodal only or parenchymal (six patients with locally extensive disease alone are excluded). Figure 3 shows the survival curves for patients with bladder and non-bladder primaries.

Patients received a median of five cycles of chemotherapy (range 1–6); 19 patients (43%) received a full six cycles of chemotherapy. Of these 19 patients only 11 (58%) had six cycles of cisplatin. One patient refused further chemotherapy after one cycle of CMV and 23 patients stopped chemotherapy prematurely because of unresponsive or progressive disease.

### *Toxicity*

Table III summarises the haematological and renal toxicity experienced by these patients. Toxicity data is not available on four patients; three died after completing the first cycle and one refused further treatment after the first chemotherapy injections. Ten patients experienced WHO grade three or four leukopenia in at least one cycle, 18 patients had grade 3 or 4 neutropenia and six patients had grade 3 anaemia with 13 patients requiring blood transfusion. Thrombocytopenia was only noted in one patient. Nausea and vomiting was experienced by the majority of patients but did not lead to the cessation of treatment in any patient with responsive disease.

Three early (and probably treatment related) deaths occurred within a few days of completing the first chemotherapy cycle. The cause of death was pulmonary oedema in two (confirmed in one by post-mortem) and gastrointestinal haemorrhage in the other. In none of these cases was neut-

**Table II** CMV chemotherapy regimen

| | | | | |
|---|---|---|---|---|
| Cisplatin | | 70 or 100 mg m$^{-2}$ | IV Days 2 | |
| Methotrexate | | 30 mg m$^{-2}$ | IV Days 1,8 | |
| Vinblastine | | 4 mg m$^{-2}$ | IV Days 1,8 | |
| (recycle day 22) | | | | |
| *Dose modifications:* | | | | |
| (1) Renal function[a]: | | | *Cisplatin* | *MTX* |
| GFR | | $>$50 ml min$^{-1}$– | 100% | 100% |
| | | 35–50 ml min$^{-1}$– | 50% | 100% |
| | | $<$35 ml min$^{-1}$– | Omit | Omit |
| (2) Peripheral blood counts[b]: | | | | |
| *WBC* | *Platelet* | *Cisplatin* | *MTX* | *Vinblastine* |
| $>$3.5 | $>$100 | 100% | 100% | 100% |
| 3.0–3.5 | $>$100 | 100% | 75% | 75% |
| 2.5–2.9 | $>$100 | 75% | 50% | 50% |
| $<$2.5 | OR $<$100 | Delay treatment at least 1 week | | |

[a]GFR = glomerular filtration rate as measured by calculated creatinine clearance. [b]All values $\times 10^9 l^{-1}$. MTX = methotrexate. WBC = total white blood count.

**Figure 1** Survival curve for 43 patients treated with CMV.

**Figure 2** Survival of patients with entirely nodal metastases or with parenchymal (lung, liver or bone) metastases.

**Figure 3** Survival of patients related to site of primary in bladder or other (renal, pelvis, ureter or prostate).

**Table III** Haematological and renal toxicity (data available on 39 patients)

| | *WHO Grade* | | | | |
|---|---|---|---|---|---|
| | *0* | *1* | *2* | *3* | *4* |
| 1. Haemoglobin | 4 | 12 | 17 | 6 | – |
| 2. Leukocyte | 9 | 9 | 11 | 8 | 2 |
| 3. Neutrophils | 8 | 1 | 12 | 9 | 9 |
| 4. Platelets | 38 | – | 1 | – | – |
| 5. Renal | 7 | 21 | 4 | 1 | – |

ropenia demonstrated, though in two, elevation of serum creatinine at the time of day 8 chemotherapy was apparent. The other two deaths occurred during the 3rd and 4th cycles respectively. One patient developed diabetic ketoacidosis (probably related to dexamethasone administration as an antiemetic) and died of gastrointestinal haemorrhage; the other died during an unexplained acute confusional state, almost certainly related to an exacerbation of chronic obstructive airways disease. These latter two deaths were not related to renal dysfunction or bone marrow suppression. None of the deaths occurred at the anticipated time of the nadir in peripheral blood counts.

### Discussion

There can be no doubt that for many patients with metastatic TCC combination chemotherapy can produce rapid and gratifying relief of symptoms associated with metastatic disease; in particular skeletal pain and leg oedema. Set against this is the undoubted toxicity of combination chemotherapy in this relatively aged population, especially when cisplatin is administered.

We have presented our experience of administering CMV chemotherapy to 43 patients with advanced transitional cell carcinoma. We have reported a complete remission rate in patients with measurable disease of 24% with an overall median survival of 7 months. The results are somewhat disappointing in such a selected group of patients. Many patients referred for treatment were too elderly or frail to receive chemotherapy and ten patients had renal impairment which precluded CMV chemotherapy.

We included ten patients in our report who did not have measurable disease. The difficulties in radiological assessment of disease in the pelvis accounted for most of these cases. Other groups have similarly included significant numbers of such patients (Harker *et al.*, 1985; Tannock *et al.*, 1989). We believe that this more accurately reflects the true patient population with advanced TCC. In comparison the M-VAC series from the Memorial Hospital included only three such patients out of a total of 133 (Sternberg *et al.*, 1989). In our series those patients with locally advanced non-measurable disease had an inferior outcome compared with the rest of the group (median survival 5 months; range 1–16 months). To have excluded them from our series would have artifically raised the median survival. Whether patients with locally infiltrative (but unmeasurable) disease in the pelvis have an inherently worse outlook than those with more distant metastases is unknown although similar observations have been made in the chemotherapy of advanced cervical carcinoma (Potter *et al.*, 1989).

Our overall results are remarkably similar to those reported by Harker *et al.* (1985) who first reported the use of CMV chemotherapy for this condition. The results are not as impressive as the M-VAC data from the Memorial Hospital (Sternberg *et al.*, 1989) but comparisons between different patient populations and treatments are not possible. In fact data on M-VAC and CMV from other centres is surprisingly sparse; one group however has been unable to reproduce the high response rates to M-VAC reported by the Memorial Hospital (Tannock *et al.*, 1989).

CMV proved a toxic regimen in our treatment population. Three early deaths were probably treatment related but no definitive proof was available. Toxic deaths have been reported in other series in approximately 2–4% of cases (Harker *et al.*, 1985; Hillcoat *et al.*, 1989; Logothetis *et al.*, 1990*a*; Sternberg *et al.*, 1989; Tannock *et al.*, 1989).

Too few randomised studies of sufficient size have been conducted to direct oncologists in the choice of treatment for advanced TCC, and most of these have been performed on the assumption that cisplatin is the superior drug for this condition. The National Bladder Cancer Collaborative Group compared cisplatin with cisplatin and cyclophosphamide in a randomised trial and 131 patients were entered (Soloway *et al.*, 1983). No significant differences in response or survival were noted between the two groups but a study of this size was unlikely to reveal anything other than large treatment differences. Similarly the Southeastern Cancer Study Group Trial (Troner *et al.*, 1987) showed no differences between cisplatin alone and cisplatin, doxorubicin and cyclophosphamide in combination (116 patients entered). Hillcoat *et al.* (1989) compared cisplatin alone with cisplatin plus methotrexate and reported no significant differences. The study was small (108 patients) and the potential for missing significant treatment differences was again noted (Scher, 1989).

A randomised study between M-VAC and CISCA (cisplatin, cyclophosphamide and doxorubicin) performed at the M.D. Anderson Cancer Center revealed a significant difference in response rates and overall median survival in favour of the MVAC group (Logothetis *et al.*, 1990*a*). A randomised comparison of cisplatin alone versus M-VAC has been reported in abstract form (Loehrer *et al.*, 1990). A significant difference in response rate (9% versus 33%) in favour of M-VAC and a 4 month prolongation of median survival was reported. Whether the absence of doxorubicin from the CMV regimen and doxorubicin and methotrexate from the CISCA regimen can explain the superior results for M-VAC in these trials is unknown. No randomised study has been performed between CMV and M-VAC.

Future directions in the management of advanced TCC remain to be defined. Accepting the toxicity of cisplatin-based chemotherapy becomes questionable when long-term survivors are rare. In the randomised studies above the overall response rates to cisplatin alone were only 16%, 20% and 31% respectively (Troner *et al.*, 1987; Hillcoat *et al.*, 1989; Soloway *et al.*, 1983). What is beyond question is that cisplatin is the drug responsible for the most toxicity. In the absence of new active agents for TCC and considering the relatively small numbers of long-term survivors after chemotherapy one approach to the problem is to attempt to minimise the toxicity of the treatment thereby making it available to more patients. With this view in mind the MRC Advanced Bladder Cancer Subgroup from Great Britain have launched a randomised trial comparing the CMV regimen (cisplatin dose 70 mg $m^{-2}$) with the same chemotherapy without the cisplatin (MV).

Even in the event that the CMV regimen were found to be superior to MV it may still be prudent to confine CMV treatment to patients with an inherently better outcome. Younger patients with good performance status could be selected for the more intensive treatment. Other prognostic factors could be utilised to determine those patients with a higher probability of achieving a complete response and possible long-term survival. In our series those patients with non-bladder primaries and disease confined to nodal metastatic sites had a significantly longer median survival. However these results should be interpreted with caution in view of the small numbers involved in the analysis. Larger trials are needed to test these prognostic factors and produce new indicators of treatment outcome.

Another approach adopted by the M.D. Anderson group is to dose intensify the chemotherapy using haemopoietic growth factors. They reported impressive response rates with escalated M-VAC chemotherapy supported by G-CSF in patients who were primarily resistant to or relapsing from combination chemotherapy (Logothetis *et al.*, 1990*b*). However platelet toxicity in that study was profound and this approach may not be widely applicable.

The results of ongoing randomised trials will hopefully provide practical recommendations for oncologists who manage patients with this common malignancy which is chemosensitive but largely incurable.

We would particularly like to thank Jill Baston for her secretarial help with this paper and Dr Pamela Smartt for her help with data analysis and survival curves.

The Wessex Medical Oncology Unit is supported by the Cancer Research Campaign.

### References

BABAIAN, R.J., JOHNSON, D.E., LLAMAS, L. & AYALA, A.G. (1980). Metastases from transitional cell carcinoma of urinary bladder. *Urology*, **16**, 142–144.

COCKROFT, D.W. & GAULT, M.H. (1976). Prediction of creatinine clearance from serum creatinine. *Nephron*, **16**, 31–41.

HARKER, W.G., MEYERS, F.J., FREIHA, F.S. & 5 others (1985). Cisplatin, methotrexate, and vinblastine (CMV): an effective chemotherapy regimen for metastatic transitional cell carcinoma of the urinary tract. A Northern California Oncology Group Study. *J. Clin. Onc.*, **3**, 1463.

HILLCOAT, B.L., RAGHAVAN, D., MATTHEWS, J. & 12 others (1989). A randomized trial of cisplatin versus cis-platin plus methotrexate in advanced cancer of the urothelial tract. *J. Clin. Onc.*, **7**, 706.

LOEHRER, P.J., ELSON, P., KÜEBLER, J.P. & 6 others (1990). Advanced bladder cancer: a prospective inter-group trial comparing single agent cisplatin (CDDP) versus M-VAC combination therapy. *Proc. ASCO.*, **9**, 132.

LOGOTHETIS, C.J., DEXEUS, F.H., FINN, L. & 4 others (1990*a*). A prospective randomized trial comparing MVAC and CISCA chemotherapy for patients with metastatic urothelial tumors. *J. Clin. Onc.*, **8**, 1050.

LOGOTHETIS, C.J., DEXEUS, F.H., SELLA, A. & 4 others (1990*b*). Escalated therapy for refractory urothelial tumors: methotrexate-vinblastine-doxorubicin-cisplatin plus unglycosylated recombinant human granulocyte-macrophage colony-stimulating factor. *J. Natl Cancer Inst.*, **82**, 667.

POTTER, M.E., HATCH, K.D., POTTER, M.Y., SHINGLETON, H.M. & BAKER, V.V. (1989). Factors affecting the response of recurrent squamous cell carcinoma of the cervix to cisplatin. *Cancer*, **63**, 1283–1287.

SCHER, H.I. (1989). Should single agents be standard therapy for urothelial tract tumors? *J. Clin. Onc.*, **7**, 694–697. (Editorial).

SOLOWAY, M.S., EINSTEIN, A., CORDER, M.P., BONNEY, W., PROUT, G.P. & COOMBS, J. (1983). A comparison of cisplatin and the combination of cisplatin and cyclophosphamide in advanced urothelial cancer. *Cancer*, **52**, 767–772.

STERNBERG, C.N., YAGODA, A., SCHER, H.I. & 9 others (1985). Preliminary results of M-VAC (methotrexate, vinblastine, doxorubicin and cisplatin) for transitional cell carcinoma of the urothelium. *J. Urol.*, **133**, 403.

STERNBERG, C.N., YAGODA, A., SCHER, H.I. & 12 others (1988). M-VAC (methotrexate, vinblastine, doxorubicin and cisplatin) for advanced transitional cell carcinoma of the urothelium. *J. Urol.*, **139**, 461.

STERNBERG, C.N., YAGODA, A., SCHER, H.I. & 14 others (1989). Methotrexate, vinblastine, doxorubicin, and cisplatin for advanced transitional cell carcinoma of the urothelium. Efficacy and patterns of response and relapse. *Cancer*, **64**, 2448.

TANNOCK, I., GOSPODAROWICZ, M., CONNOLLY, J. & JEWETT, M. (1989). M-VAC (methotrexate, vinblastine, doxorubicin and cisplatin) chemotherapy for transitional cell carcinoma: the Princess Margaret Hospital experience. *J. Urol.*, **142**, 289–292.

TRONER, M., BIRCH, R., OMURA, G.A. & WILLIAMS, S. (1987). Phase III comparison of cisplatin alone versus cisplatin, doxorubicin and cyclophosphamide in the treatment of bladder (urothelial) cancer: a Southeastern Cancer Study Group trial. *J. Urol.*, **137**, 660.

YAGODA, A. (1980). Chemotherapy of metastatic bladder cancer. *Cancer*, **45**, 1879–1888.


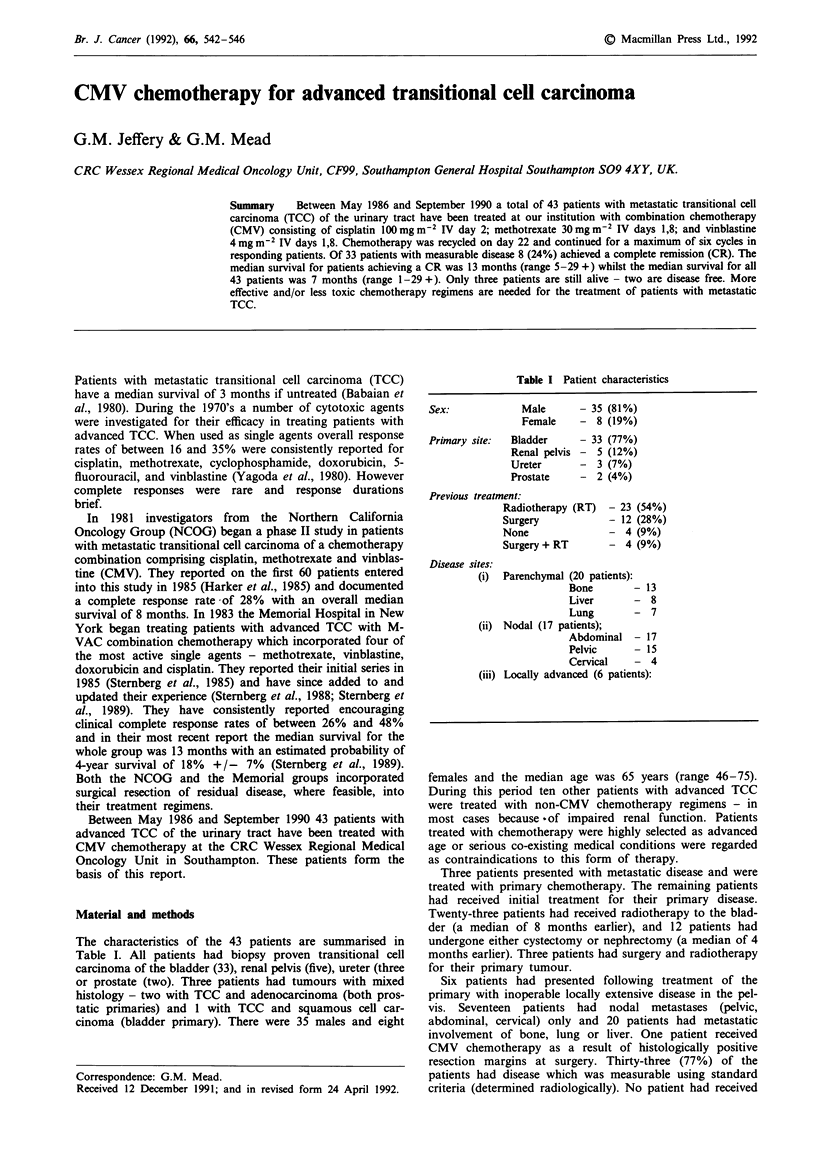

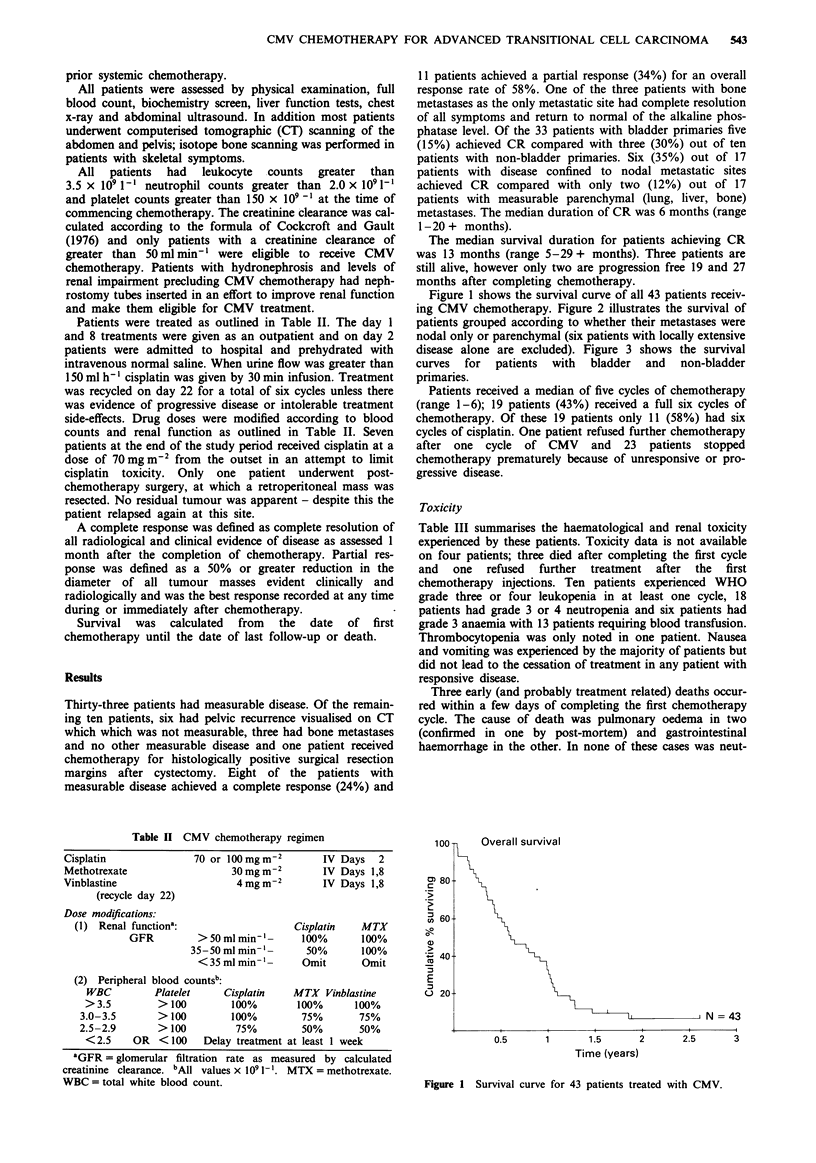

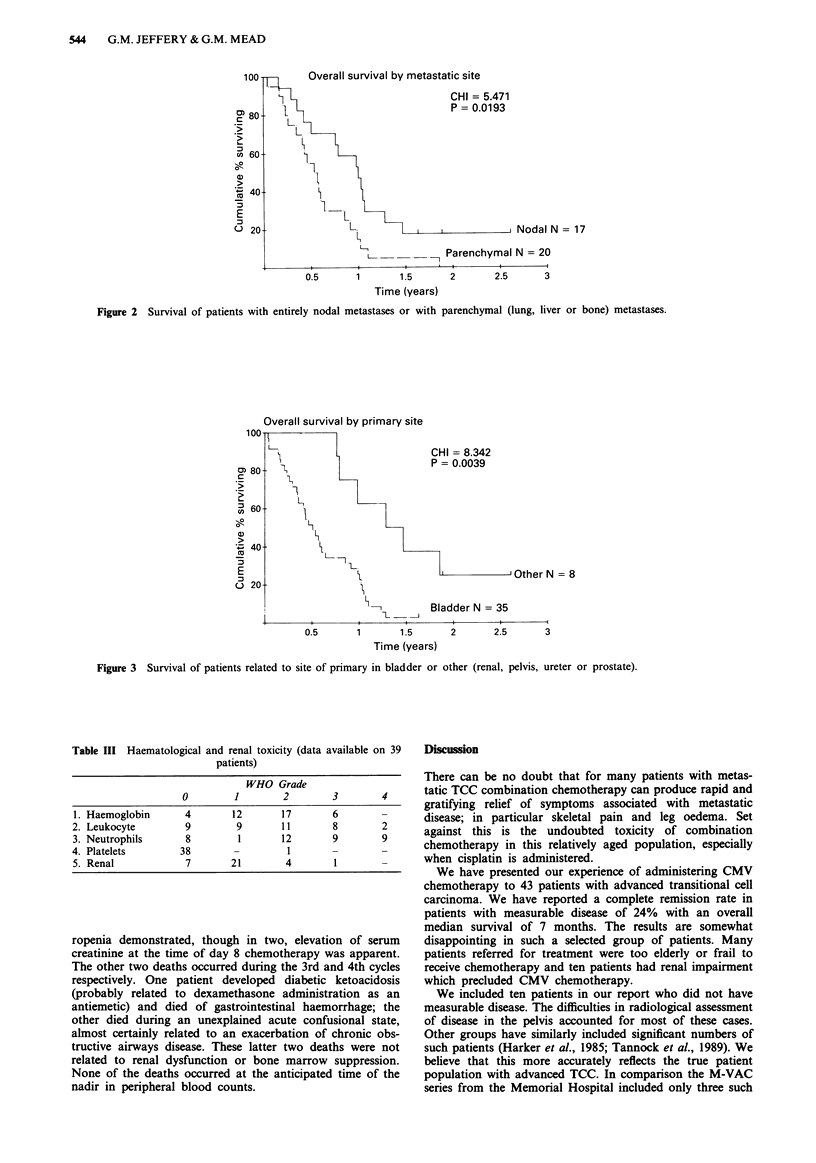

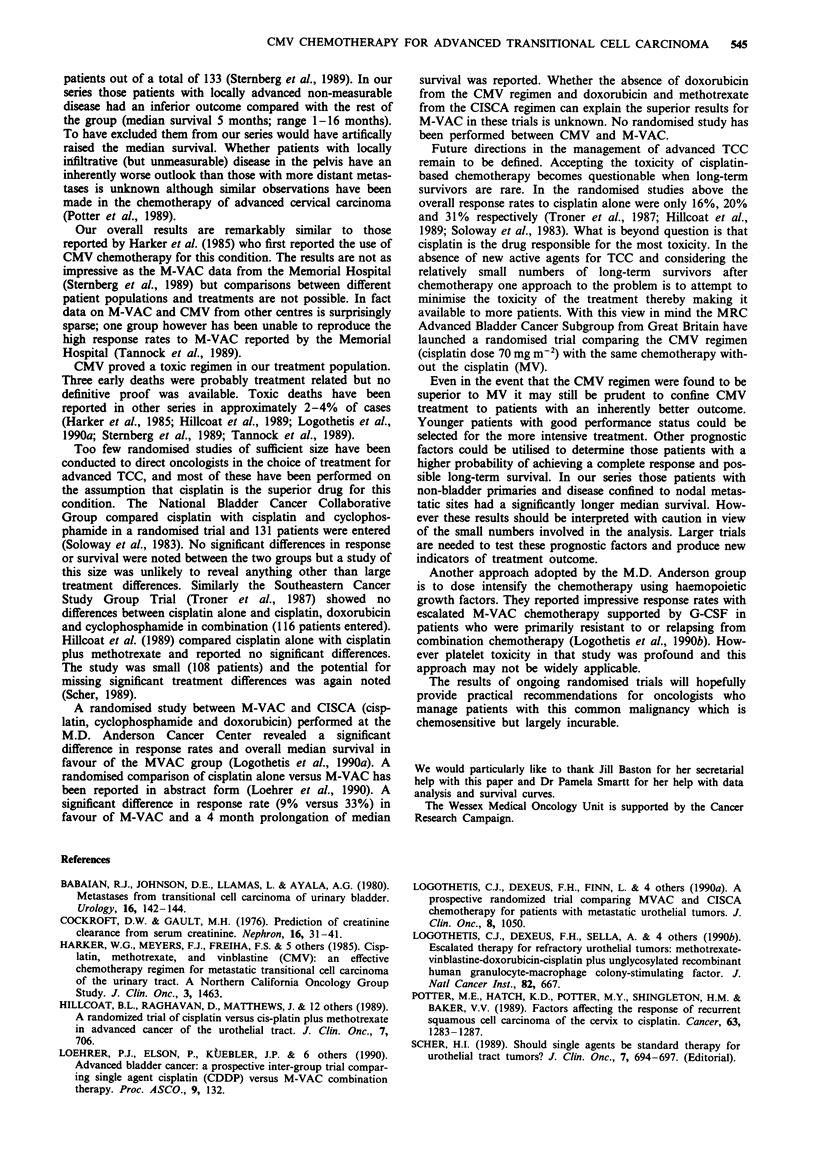

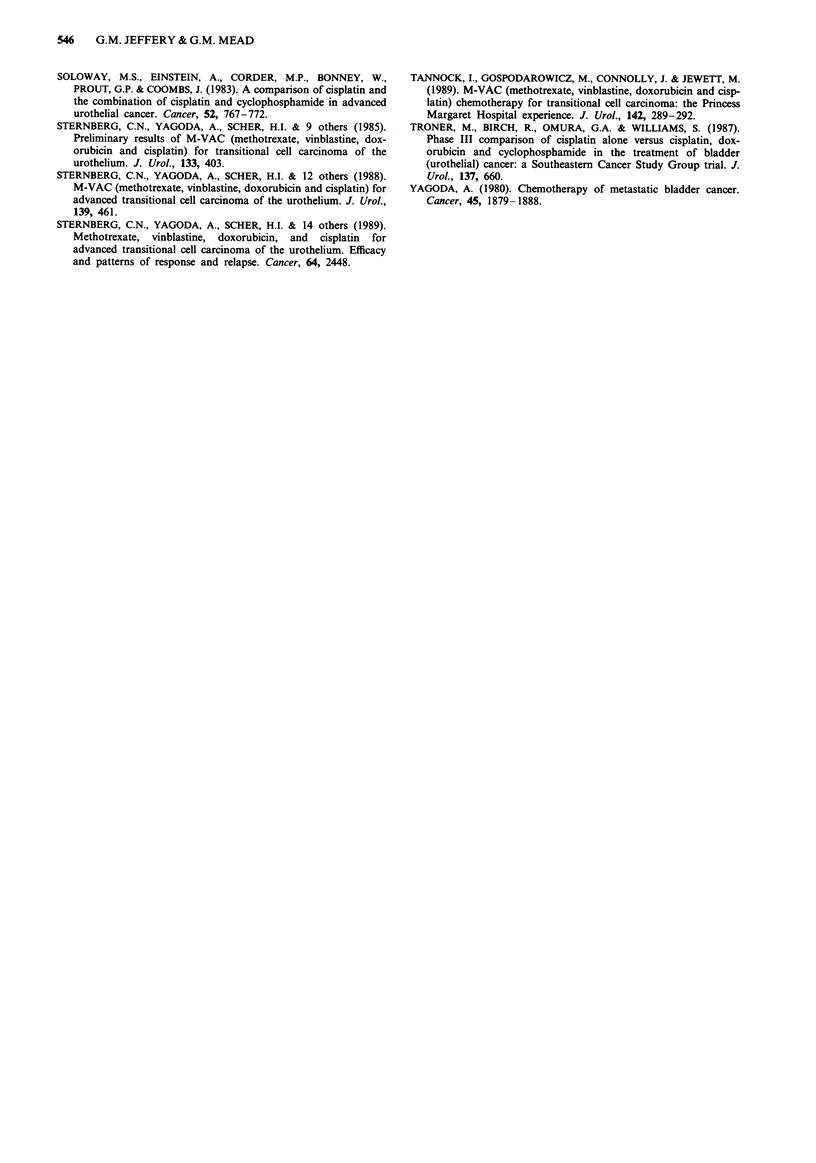


## References

[OCR_00559] Babaian R. J., Johnson D. E., Llamas L., Ayala A. G. (1980). Metastases from transitional cell carcinoma of urinary bladder.. Urology.

[OCR_00564] Cockcroft D. W., Gault M. H. (1976). Prediction of creatinine clearance from serum creatinine.. Nephron.

[OCR_00570] Harker W. G., Meyers F. J., Freiha F. S., Palmer J. M., Shortliffe L. D., Hannigan J. F., McWhirter K. M., Torti F. M. (1985). Cisplatin, methotrexate, and vinblastine (CMV): an effective chemotherapy regimen for metastatic transitional cell carcinoma of the urinary tract. A Northern California Oncology Group study.. J Clin Oncol.

[OCR_00577] Hillcoat B. L., Raghavan D., Matthews J., Kefford R., Yuen K., Woods R., Olver I., Bishop J., Pearson B., Coorey G. (1989). A randomized trial of cisplatin versus cisplatin plus methotrexate in advanced cancer of the urothelial tract.. J Clin Oncol.

[OCR_00587] Logothetis C. J., Dexeus F. H., Finn L., Sella A., Amato R. J., Ayala A. G., Kilbourn R. G. (1990). A prospective randomized trial comparing MVAC and CISCA chemotherapy for patients with metastatic urothelial tumors.. J Clin Oncol.

[OCR_00593] Logothetis C. J., Dexeus F. H., Sella A., Amato R. J., Kilbourn R. G., Finn L., Gutterman J. U. (1990). Escalated therapy for refractory urothelial tumors: methotrexate-vinblastine-doxorubicin-cisplatin plus unglycosylated recombinant human granulocyte-macrophage colony-stimulating factor.. J Natl Cancer Inst.

[OCR_00600] Potter M. E., Hatch K. D., Potter M. Y., Shingleton H. M., Baker V. V. (1989). Factors affecting the response of recurrent squamous cell carcinoma of the cervix to cisplatin.. Cancer.

[OCR_00606] Scher H. I. (1989). Should single agents be standard therapy for urothelial tract tumors?. J Clin Oncol.

[OCR_00612] Soloway M. S., Einstein A., Corder M. P., Bonney W., Prout G. R., Coombs J. (1983). A comparison of cisplatin and the combination of cisplatin and cyclophosphamide in advanced urothelial cancer. A National Bladder Cancer Collaborative Group A Study.. Cancer.

[OCR_00620] Sternberg C. N., Yagoda A., Scher H. I., Watson R. C., Ahmed T., Weiselberg L. R., Geller N., Hollander P. S., Herr H. W., Sogani P. C. (1985). Preliminary results of M-VAC (methotrexate, vinblastine, doxorubicin and cisplatin) for transitional cell carcinoma of the urothelium.. J Urol.

[OCR_00630] Sternberg C. N., Yagoda A., Scher H. I., Watson R. C., Geller N., Herr H. W., Morse M. J., Sogani P. C., Vaughan E. D., Bander N. (1989). Methotrexate, vinblastine, doxorubicin, and cisplatin for advanced transitional cell carcinoma of the urothelium. Efficacy and patterns of response and relapse.. Cancer.

[OCR_00624] Sternberg C. N., Yagoda A., Scher H. I., Watson R. C., Herr H. W., Morse M. J., Sogani P. C., Vaughan E. D., Bander N., Weiselberg L. R. (1988). M-VAC (methotrexate, vinblastine, doxorubicin and cisplatin) for advanced transitional cell carcinoma of the urothelium.. J Urol.

[OCR_00636] Tannock I., Gospodarowicz M., Connolly J., Jewett M. (1989). M-VAC (methotrexate, vinblastine, doxorubicin and cisplatin) chemotherapy for transitional cell carcinoma: the Princess Margaret Hospital experience.. J Urol.

[OCR_00642] Troner M., Birch R., Omura G. A., Williams S. (1987). Phase III comparison of cisplatin alone versus cisplatin, doxorubicin and cyclophosphamide in the treatment of bladder (urothelial) cancer: a Southeastern Cancer Study Group trial.. J Urol.

[OCR_00649] Yagoda A. (1980). Chemotherapy of metastatic bladder cancer.. Cancer.

